# Elements of General Anatomy, Containing an Outline of the Organization of the Human Body

**Published:** 1830-01-01

**Authors:** 


### Elements of General Anatomy, Containing an Outline of the Organization of the Human Body. By *R. D. Grainger*, Lecturer on Anatomy and Physiology. Pp. 526.

Although the study of minute anatomy has been cultivated with some assiduity since the days of Berenger and Vesalius, the progress made in its elucidation was comparatively trifling until the accurate dissections of Ruysch and Malpighi and the indefatigable investigations of Haller invested it with the size and dignity of a leading science. The ancient doctrine of four elementary constituents continued to be taught in the schools of medicine for many centuries after its original proposer had been removed; chemistry was studied under so many disadvantages that little could be expected from her discoveries; and anatomy was so much confined to the inspection of inferior animals, and so encompassed with silly theories, that the structure of the human body was but slowly developed. Even Haller himself, who was in talent and information at least a century before his age, was sadly trammelled with the fancy that there existed a rudimental fibre, into which every form of animal substance might be reduced; and, although this fibre was admitted to be invisible, "invisiblis ea fibra," he could not avoid hazarding many fanciful hypotheses as to its properties and composition. Cullen and the Hunters in this country, Bonn at Amsterdam, and Pinel, in France, built, with very considerable success, upon the foundation which had been laid; and two years after the *Nosographie Philosophique* of the last author made its appearance, Xavier Bichat presented the world with the first unique and consistent view of the general structure of the animal tissues. The arrangement laid down by this distinguished anatomist it is unnecessary to detail; suffice it to observe that in the subsequent classifications of Dupuytren, Richerand, Cloquet, Meckel, Mayer, and Beclard it has been carefully consulted, and while greater facilities for anatomical research and more extensive inquiries have enabled some of his followers to improve upon his plan, the general ground-work of all their systems is essentially the same.

Although England has contributed her full share to the advancement of minute anatomy, nothing meriting the title of a system existed in this country before the appearance, in 1828, of Dr. Craigie's laborious and instructive work. To remedy this defect, Mr. Grainger has published the present volume, and although the "Elements of General and Pathological Anatomy" of the Scotch metropolis have been devoted to a majority of the same subjects, there is, notwithstanding, ample room for both, more especially when it is considered that rudimental structure is taught in Dr. Craigie's Elements with a reference to pathology, while physiology is the subject which the present author has had more immediately in view during his illustrations of animal texture.

The human body consists of solid and fluid matter variously arranged and curiously modified in external character. These two forms of matter exert upon each other considerable influence. The solids are built up by the consolidation of the fluids, the fluids depend for their circulation and consistency upon the action of the solids, and most of the fluids are elaborated by solid organs. Diseases of the one class are easily communicated to the other, and neither can continue in a state of health for any length of time without mutual participation.

"In studying the wonderful structure of our frame, we should observe the same method as in investigating the nature of any other material body: that is to say, we should endeavour to separate the fluid from the solid parts; we should examine these individually, in order to ascertain their properties, their differences, and their uses; in fact we should analyse, as far as this is possible, the animal body, so as to determine the nature of its constituent parts. This mode of procedure is well calculated to unravel the intricacy of the human organization; but in order to complete our knowledge of the disposition and operations of this complicated machine, it is necessary to reverse the method of examination; to trace, in a synthetic order, the various combinations of the fluid and solid materials; to study the properties and uses of the different organs; and, lastly, to compare the relations that exist between them, so that the share which each enjoys in the production of the vital phenomena, may be properly distinguished.

"The researches of animal chemistry, to which anatomy and physiology are so deeply indebted, have shewn that the human body is composed of a great number of elementary substances, which are termed its ultimate principles. The exact number of these simple materials has not yet been determined, but the following are generally admitted:—

ELEMENTARY SUBSTANCES.

| | |
|---|---|
| 1. Azote. | 9. Potassium. |
| 2. Carbon. | 10. Magnesium. |
| 3. Oxygen. | 11. Iron. |
| 4. Hydrogen. | 12. Chlorin. |
| 5. Phosphorus. | 13. Manganese? |
| 6. Calcium. | 14. Silicium? |
| 7. Sulphur. | 15. Fluorin? |
| 8. Sodium. | 16. Iodin? |

"The combination of these elements in different forms and proportions, produces certain compounds which are called the immediate or proximate principles of formation. They vary considerably according to the age of the individual, the state of health, and other circumstances; so that it is very difficult to ascertain what number really belongs to the composition of the healthy body.

PROXIMATE PRINCIPLES.

| | |
|---|---|
| 1. Gelatin. | 6. Mucus. |
| 2. Albumen. | 7. Urea. |
| 3. Fibrin. | 8. Picromel. |
| 4. Colouring Matter of the Blood. | 9. Sugar of Milk. |
| 5. Fatty Matter .. { Olein. Stearin. Cholesterin. | |

ACIDS.

| | |
|---|---|
| 1. Phosphoric | 7. Oxalic. |
| 2. Uric. | 8. Acetic. |
| 3. Carbonic. | 9. Butyric? |
| 4. Sulphuric. | 10. Purpuric? |
| 5. Muriatic. | 11. Malic?" 24. |
| 6. Benzoic. | |

The application of the microscope to minute anatomy is generally supposed to have been productive of much good; but the conflicting results which have issued from microscopical inquirers, equally celebrated for their integrity and acuteness, cannot fail to cast an aspect of suspiciousness and uncertainty upon all their observations. Meckel reduces the ultimate animal constituents to two, one of which is globular and enters freely into the composition of many of the fluids; the other is susceptible of coagulation, and when coagulated forms all the solids, when liquid, the great body of the fluids. The globules are never seen alone, but always floating in the coagulable fluid. They vary in size, form, number and color in different organs, at different ages, and in different individuals. In the blood they appear to consist of a solid centre, and a surrounding vesicle, which encloses, but is not attached to it. They are smaller in the liver than the spleen, in the nerves than the blood, and they are larger in the blood than in the lymph, milk, or chyle. They are numerous in muscle and nerve; absent from bone, cartilage, and fibre; more numerous in the blood than in chyle, or milk, and their color varies with the organ of which they constitute a part. Dr. Edwards, of Paris, asserts that the elementary structure of the cellular, fibrous, muscular, vascular and nervous tissues is globular, that the globules are spherical, each having a diameter of $\frac{1}{300}$ of a millimeter, and that they agree in form and size in whatever organ, or animal they may be found; while Dr. Hodgkin disagrees with both Meckel and Edwards. He contends that Hewson, Home, Meckel, Banes, and Prevost have erred, in supposing that the elementary particles of the blood consist of a central globule, and an external vesicle. According to his observations they appear like *flattened cakes*, which are nearly or wholly colourless, have thick and rounded edges, and are depressed in the centre. He even denies *in toto* the existence of globules in the muscular, nervous, and cellular tissues —the very tissues which were referred to by others as more strikingly illustrative of globular structure, and he has discovered that the fibres of the muscular tissue are marked by transverse lines, which impart an appearance peculiar to themselves.

By these observations we mean not to cast discredit upon the information of the microscope; our object is merely to enter our *cavete* upon the records of minute philosophy, and to warn its advocates from obvious and prolific sources of disappointment. If elementary structure can be made more uniform, if the laws of organization can be shewn to be more simple, if it can be proved that animal texture is rudimentally the same wherever it occurs, then, in the name of science, proceed in the cultivation of microcosmic knowledge. But the glaring discrepancies which have already marked the microscope tempt us to take a weighty per centage off the conclusions it announces, and the palpable tendency to deception which it is obnoxious to, make us look upon its employment with some misgiving and a degree of doubt. Young asserts that the size of the red particles of the blood is $\frac{1}{6060}$ part of an inch, while Hodgkin makes it $\frac{1}{3000}$, or not half so large; Wollaston concludes it to be $\frac{1}{4000}$, while Banes makes it $\frac{1}{1700}$; and Turine estimates it at $\frac{1}{5240}$ at one measurement, and only $\frac{1}{1940}$ at another. Whether such inconsistencies arise from our attempting minutiæ for which we have no instruments, or whether they depend upon the deceptiveness of the instruments we have, are questions which it may perhaps in the present condition of the subject be more useful to propose than attempt to answer.

Following the example of Chaussier the author arranges the fluids of the human body into four classes—1st, the fluids which form the blood, 2dly, the blood formed by these fluids, 3dly, the fluids formed by the blood, and 4thly, the fluids returned to the blood. This division may be seriously objected to by anatomists, who would base their arrangement upon structure, or by chemists who would erect it upon analysis, but to physiologists it must be especially appropriate, as it sacrifices every thing to accord with function; and being comprehensive enough to embrace every fluid, as well as distinctive enough to individualize them, the most important objects of an arrangement are attained. Of all the animal fluids by much the most important is the blood, whether its quantity, properties, or uses be considered. Harvey calculated that the blood constituted one-twentieth of the entire body, and Keil went so far as to estimate its quantity at 100 pounds. Its ordinary temperature, which is 98°, may be greatly altered by disease. In the cold fit of ague it has fallen to 94°, and in tetanus it has risen to 110°. About three minutes and a half after its removal from the body it begins to coagulate, and in seven its coagulation is generally complete. When the quantity drawn is small it coagulates sooner than when large, and arterial is slower in coagulating than venous blood. According to Mr. Thackrah the strength or tone of the system is in an inverse ratio to the time occupied by the coagulation of the blood. When coagulation is slow the strength is great; when quick it is feeble. Hence blood, which is drawn during active inflammation, will remain much longer fluid than healthy blood, or blood drawn from a debilitated habit; and for the same reason the first cup which is filled from a vein will coagulate more slowly than the last. These are observations of the most interesting nature both in a physiological and practical sense, and their intrinsic interest should be heightened by the fact that their accuracy cannot be the subject of dispute. It was John Hunter who first extricated the blood from the catalogue of dead fluids, and maintained that it was as largely endowed with life as nerve or muscle; and although the strong holds of prejudice for a time refused admission to such a doctrine, and although the natural abhorrence felt by every mind against important innovations earnestly wrestled with the arguments by which it came supported, every fresh investigation has only proved a fresh confirmation of its truth, and now there should not remain either difficulty or doubt upon the question. Compare the blood with any other fluid, (except the chyle, which is blood in its first stage,) oppose property to property, contrast phenomenon with phenomenon, and where are the common points, the generic similarities by which they are connected? If the blood be a lifeless fluid like the saliva, or bile, or urine, it ought to present the same properties and appearance in the cup as in the vein. Why does it continue fluid while connected with the system? Why does it coagulate when it leaves it? Why does its coagulability increase as vital power diminishes, and diminish as it increases? Why does it get sickly and diseased when the general system is disordered? Why does it deposit living muscle, living vessel, and living nerve, if it be itself lifeless; and why should its removal from the body lower life in proportion to the quantity removed? Will bile separate into its constituent parts when removed from the gall-bladder? Can it be killed with a stroke of lightning? Can it be inflamed like a piece of muscle? If a muscular fibre or nervous filament be separated from the centre of life they instantly die, speedily decompose, and ultimately putrify; and if a few globules of blood be taken from an artery or vein they experience the same changes. Where then are the facts which shew that the blood is a lifeless fluid? If its dissimilarity to dead and its resemblance to living matter can be any indication of its own vitality both are equally well established. Sir A. Cooper and Mr. Thackrah have maintained that the blood-vessels impart a vital influence to their contents, in virtue of which they continue fluid as long as they remain in contact with the vessels in a healthy state; and that the moment they are removed from the living tube they lose their fluidity and coagulate. But it is rather singular that two inquirers of such acumen could have overlooked one link in this chain of reasoning which is so weak as to incapacitate the whole. If the blood be preserved fluid by a living principle communicated to it by the vessel, it is absurd to suppose that it is itself dead; otherwise the nervous influence can imbue dead matter with living principle, while that same matter, to which the living principle is imparted, continues destitute of life! On this point Mr. Grainger observes with much judgment—"I think it will be admitted, that the blood must itself be endowed with life, in order that it may be capable of being affected by the nervous influence of its containing vessels; for it is self-evident, that no fluid deprived of vitality can receive any impression from simply being introduced into a living vessel." 45.

In the rude and shapeless embryo the solids bear a smaller proportion to the fluids than they do in the adult; and yet in the full grown body, where organization has reached its highest point, they do not seem to compose much more than one-sixth of the whole fabric. Chaussier desiccated a body weighing 120 pounds for several days, and at the end of the experiment its weight was reduced to 12 pounds; and an adult mummy was found to weigh only seven pounds and half.

"The ancients supposed that every solid organ might be reduced by a species of anatomical analysis, to a simple fibre, which they called the elementary fibre. They said, that this was every where of the same nature, and that it was formed of earth, oil, and iron. They also imagined, that this fibre produced what we term the cellular tissue; and that this tissue, under different degrees of condensation, formed the various organs of the body. Haller, who admitted the existence of the elementary fibre, thought that it was not visible, and that it could only be distinguished by the mind; being to the anatomist, what the line is to the geometrician. This opinion is evidently erroneous, as matter must have existence. It is equally incorrect, at least in man and in the superior animals, to imagine that the cellular tissue forms the basis of all the solids. This tissue is, undoubtedly, the most extended element in the animal organization; but it is not the only one. The best anatomists of the present day are agreed, that there are at least three, if not four, elementary fibres, viz. the cellular, the muscular, and the nervous. This division, which was suggested by Haller and adopted by Blumenbach, has been modified by Professor Chaussier, who contends, that there is a fourth fibre, which he calls the albugineous. Many distinguished authorities, in whose opinion I concur, believe this to be merely a condensed modification of the cellular fibre." 58.

These elementary tissues are not equally extensive, nor important. In many animals nothing but cellular texture is discoverable, and in some no nervous matter has as yet been found. Variously combined and proportioned the cellular, muscular, and nervous tissues give origin to a great variety of compound solids, and these solids are furnished with properties adapted to the places they occupy and the functions they discharge.

Bichat was the first, who endeavoured to reduce into a digested system the different forms which organic solids assume, and the arrangement which he made, while it is in general too minute and in some parts deficient, has furnished subsequent and much less industrious writers with materials for new and more accurate divisions. To separate arteries from veins and exhalants from absorbents, to divide the nervous system into two departments to accord with his distinction of animal and organic life, to make the synovial tissue different from the serous, and the pilous from the epidermoid, was, certainly, making many differences out of few distinctions, and overcharging what might otherwise have been a simple catalogue. The following is Mr. Grainger's plan, which is not very different from that of Mayer.

| | | | |
|---|---|---|---|
| " 1. Cellular. | | 6. Cartilaginous. | |
| 2. Serous. | | 7. Fibrous. | Fibrous.<br>Fibro-cartilaginous |
| 3. Cutaneous. | Dermoid.<br>Mucous. | 8. Osseous. | |
| 4. Vascular. | Arterial.<br>Venous.<br>Lymphatic.<br>Erectile. | 9. Muscular. | Voluntary.<br>Involuntary. |
| | | 10. Nervous. | Cerebral.<br>Ganglionic. |
| 5. Glandular. | | 11. Epidermoid. | |

" These different systems are united and combined together in order to form the organic apparatuses, which may be divided according to their functions, into three classes.

" I. The process of nutrition is accomplished by the following apparatuses :—

" *a.* The apparatus of digestion, consisting of the alimentary canal, extending from the mouth to the anus, with its numerous appendages, such as the teeth, the salivary glands, the liver, &c.

" *b.* The apparatus of respiration, comprising the lungs, with the organs which are subservient to their action, as the bones and muscles of the thorax, the wind-pipe, &c.

" *c.* The apparatus of circulation, comprehending the heart, the arteries, the veins, and the lymphatics.

" *d.* The apparatus of secretion, formed of the glands, the follicles, and the perspiring surfaces. Several of these organs also assist in other functions, and are comprised in their apparatuses ; thus, the liver purifies the blood by the secretion of the bile, and at the same time, by that fluid, assists in the digestive process.

" II. The apparatuses of external relation comprehend :

" *a.* The apparatus of sensation, which includes the skin and the organs of the other senses.

" *b.* The apparatus of motion, formed of the bones, the ligaments, the muscles, and the synovial bursæ.

" *c.* The apparatus of the voice, consisting of the cartilages and muscles of the larynx, and also of the organs of speech.

" *d.* The apparatus of perception and volition, which is composed of the brain and nerves.

" III. The apparatus of reproduction consists of:

" *a.* The organs of generation in the male, including the testes and penis.

" *b.* The organs of generation in the female, comprising the uterus and the ovaria, and their appendages." 71.

The degrees by which these several tissues arrive at perfection are many and minute. When the ovum is examined upon the 8th day it appears to be composed of two membranes. The first or external membrane is open throughout its length; the second or internal one contains a slimy fluid and two vesicles. At this period no globules can be detected, after some time they appear in trifling quantity, and at a more advanced stage they are seen to unite so as to form fibres. It was once believed that organization proceeded from the centre of an organ towards its circumference, but the reverse is now found to be the fact, and it is not less curious that at first every organ is double, and that the duplicates are afterwards united. The spinal cord and brain arise by two plates, and even the intestinal canal is not cylindrical until its two halves are united. The relics of this symmetrical arrangement are very obvious even in the adult. The raphe which divides the perineum, the mediastinum which separates the lungs, the fossa which marks the upper lip, the septum of the nostrils, and the falx between the hemispheres of the brain are evidently the remains of a grand longitudinal division; and the cleft palate, the hare lip, and the divided perineum seem to depend for their formation upon this principle.

During every period of life the human body is changing its form, and varying its dimensions. At birth the head and nervous system, the heart and vascular system, preponderate very materially over the intestines, spleen, stomach and lungs; at puberty the organs of generation are developed; at maturity organization is perfect, and every function has reached its most finished state; and at old age the fluids become scanty, the solids unyielding, and function languid. These successive alterations in the organization of the body are accompanied with considerable changes in the energies of mind and in the condition of life. While the fluids are in excess during infantile growth impressions are easily made and dissipated, the mind is lively but uncertain, the passions are ardent but speedily cooled, the body is energetic but soon exhausted. Between puberty and manhood the mental powers are in their most vigorous exercise, every organ is firmly braced about with strength, and every function is punctually discharged; and as the body descends the vale of years imagination languishes, judgment becomes weak, memory faithless, and manhood sinks into a second childhood.

This statement, as a general one, is correct, but many brilliant exceptions are occurring daily—exceptions which ought to moderate the language of materialists. If organization be the cause and not the concomitant of life, the effect should in all instances be proportionate to the cause; mind should sink and rise with corporeal vigour; dilapidated constitutions should be as remarkable for pusillanimity as for weakness; healthy systems should be found in the Homers of the age, and the flame of genius should be seen sinking in company with the taper of life. But as an universal principle how far is the case otherwise? Our authors of the widest caliber are too often afflicted by disease, or harrassed by debility; some of the most brilliant effusions, which ever genius poured forth, issued from men who had far passed the zenith of life; how many hoary headed veterans of scienee in the present day could be enumerated, whose mental powers are standing firm during the wreck and ruin of every organ; and how many spirits, resisting the combined influence of sickness and of age, have at the close of a splendid career rather ceased to burn within fabrics, which had been rendered unfit for their occupation, than been buried amid their ruins!

The external characters of the body are materially influenced by the state of the sexual function; and any derangement in the construction, or deficiency in the development of the genital apparatus is almost universally attended with some constitutional peculiarity.

"Castration will even, to a certain extent, produce in one sex the characters of the other: the ovaries were removed in a woman at Bartholomew's hospital; she afterwards grew thinner and more muscular; her breasts shrunk away, and she ceased to menstruate. The absence of the uterus only, does not prevent the individual from acquiring the general characters of the sex, nor does it destroy desire.

"The changes produced by the imperfection of the genital organs, is still more remarkable in some birds. It was noticed by Mr. Hunter, that the female bird sometimes acquires a plumage considerably resembling that of the male, and he supposed that this metamorphosis was connected with the age of the bird, and that it only occurred when she ceased to lay. Mr. Yarrell, who has presented a paper to the Royal Society, on the change of plumage which the hen-pheasant occasionally experiences, has found that it does not depend on age, but that it is connected with some disease or imperfection of the sexual organs. He further observes, that when these organs are imperfect in either male or female, the sexes approximate so much, that it is difficult to distinguish between them." 88.

We have now taken some notice of the principal points which the author has thought proper to comprehend within his introduction, and the reader can scarcely fail to be struck with their number and importance. Considering the nature of the present work, as well as the form of its title-page, we believe it would have been more beneficial to both had many of these preliminary observations been thrown into the body of the performance, and worked up into a distinct chapter. In an elementary work of this nature, one-fifth of the volume will, we have no doubt, be considered much more than enough for prefatory remarks, and when many, if not the most of these remarks are as strictly part and parcel of the general subject as are any of the topics discussed in the succeeding pages, it would have been not only more methodical, but in every sense more useful to have limited the introduction to the first seventeen pages, which alone are properly introductory, and to have opened the first chapter with the fourth section. Although we do not live in the days of the humoral pathology, and cannot discover as much disease and death in the animal fluids as our ancestors did, yet they should neither be wholly overlooked, nor hastily passed over. They are a most important department of the animal machine, they have laws and properties of their own which should be understood; they have a structure which ought to be unravelled, and they are not merely subject to disorder but frequently to disease. If, therefore, their natural properties and healthy anatomy be unknown, it is not less difficult to recognize derangement in them, when in an unhealthy state, than in a muscle or a membrane, if ignorant of its ordinary characters. Now, in the Elements of General Anatomy, the solids alone occupy the body of the work; the fluids are entirely neglected. In the introduction, it is true, the chemistry of the chyle, blood and lymph is given, something is said of their elementary composition, and a short table of the animal fluids generally is laid down; but few readers will think of looking into the introduction for matter which should hold a conspicuous place in the body of the book, and those who may be induced to do so in consequence of missing it elsewhere, will probably feel with us that it neither occupies its proper place nor receives the consideration it deserves. Influenced by this impression we have devoted more time to the introduction than we should otherwise have done, and in now proceeding to the *first chapter* we feel as though we were passing to the *second part.*

*Cellular Tissue.* Of all the tissues the cellular is the most important, because the most abundant in quantity and the most extended in situation. Haller first demonstrated that it was continuous over the body, that it was the connecting medium between different and distant organs, and that it entered largely into the composition of most of the solid viscera. Being of a very elastic nature it exists in great profusion around joints, to facilitate their movements; it is more abundant upon the anterior than posterior surface of the body, to enable a more extensive forward than backward motion, and it surrounds all the large viscera as the kidneys, pancreas and liver. Sometimes it is quite soft and loose, at others it is condensed into hardened fasciæ. It forms the mucous, serous and fibrous membranes; with the exception of the middle coat it forms arteries and veins; lymphatics and lacteals are composed of it; under the name of parenchyma it constitutes the greatest share of secreting and absorbing glands; it envelops every nervous fibrilla and every muscular filament. According to Haller the cellular tissue is composed of numerous laminæ, which, by crossing each other in every direction, form cells of various shapes and sizes. But this opinion was controverted by Borden, who maintains that it is an amorphous semi-solid, not unlike to animal jelly; and many of the most distinguished physiologists accord with this view. Meckel more especially is its strong abettor, and the fibrous texture together with the cellular appearance which this tissue certainly assumes under the microscope, depend, he asserts, upon its being stretched out into an unnatural state and upon the entrance of air within its cells while it is extended.

"From the examinations which I have made of the cellular membrane, both with the naked eye and with the aid of an excellent microscope, I am induced to believe, that it consists of an immense number of fibres which cross each other in every possible direction, and thus intercept very irregular spaces. I have not been able to detect any *linear arrangement* of globules, although rounded corpuscles may be seen at irregular distances, which are in some places clustered together, and in others dispersed in an isolated manner. It is difficult to determine whether these globules consist of solid matter, or merely of an animal fluid. When the cellular tissue is viewed through the microscope, nothing but fibres can be seen; so that it appears the layers, which are apparent to the naked eye, consist merely of fibres joined together.

"The cells, which have no determined form or size, communicate with each other. Among the many proofs which might be adduced in support of this position, the most decisive is the general diffusion of air and fluids when they have been extravasated. Thus in emphysema, the whole of the body has been seen enormously distended with the air which has escaped from the wound of the lung.* In a similar manner, butchers inflate animals, by making a puncture in some part where the tissue is lax, and from that one aperture the air is forced to the most distant parts of the body. In anasarca the fluid gravitates to the most depending situation; and in slighter affections, induced by general debility,

* "A very remarkable case of emphysema is related by Dr. W. Hunter, Med. Obs. and Inq. vol. ii. p. 17, *et seq.*"

any effusion which happens, is seen in the legs, especially about the ankles. Again, in rupture of the bladder or urethra, the urine is extravasated in the perineum, scrotum, penis, and even thighs. Those who deny the fibrous and cellular structure of this tissue, refer the above phenomena to the permeability which it possesses, owing to its soft consistence." 120.

Haller believed that the cellular fibre consisted of earthy particles connected by an intermediate cement, composed of oil and water; Boerhaave, Ruysch and Mascagni that it was a small vessel; but more modern inquiry has ascertained that it consists of albumen, jelly and animal mucus. Cellular tissue so resists putrefaction that it may be immersed for months under water without being decomposed. It is very cohesive, flexible and elastic; in a healthy state quite insensible, and, although painful when inflamed, it is not easy to determine whether the pain depend upon any newly-acquired sensibility, or over distention of the nerves which traverse it.

*Adipose Tissue.* In addition to the cellular texture now described there is an adipose tissue, which only differs from it in function. It consists of small oval cells or vesicles, which are quite distinct from each other and from the common cells, which are not supposed to form the seat of dropsy, nor to be filled with air in emphysema, They vary in size from $\frac{1}{600}$ to $\frac{1}{300}$th part of an inch, and their principal use is as a reservoir for containing animal oil, from which nutriment may be derived when any thing occurs to obstruct or arrest the digestive function.

"This is strikingly illustrated in hibernating animals, which are very fat when they fall into the torpid state, but at the return of spring, they are much reduced in bulk. A very curious instance of life being supported by the absorption of the fluids of the body, occurred some years since at Dover. A hog weighing one hundred and sixty pounds, was buried under a portion of the cliff, which fell on its stye, for the long space of one hundred and sixty days. At the end of this time, being dug out, it weighed only forty pounds, and was extremely emaciated, clean, and white. As there was neither food nor water in the stye when the cliff fell, this hog must have existed during the time mentioned, by the removal of the adipose and other fluids from their containing structures, into the circulating system." 139.

*Serous Tissue.* The serous tissue forms membranes which have no communication with each other, and which with one exception always assume the shape of shut sacs,—*sacs sans ouverture.* These sacs are sometimes very simple, as in the bursæ mucosæ, which are merely large vesicles; but frequently they are more complex. When these membranes surround solid organs, as the heart, they are sacs so reflected upon themselves that the reflected portion covers the organ they enclose, so that when the organ is removed or the reflected portion detached a simple sac is obtained. These sacs cannot, therefore, be said to *contain* the organs which they cover, for they are attached to them by their exterior and not interior surface; and in strict language it cannot be said that the heart is within the pericardium, nor the lungs within the pleura, nor any of the abdominal viscera within the peritoneal sac. Like the cellular tissue, the serous membranes are frequently met with in those parts of the body which are subject to pressure, or motion. Thus bursæ mucosæ are found around the patella, olecranon, tendons, wrist, and finger-joints. A large bursa, or as it is more generally called capsula synovialis, enters into every joint and is reflected over the articular cartilages, capsular and lateral ligaments. Dr. Gordon and Magendie have denied that the synovial capsule is continued over the articular cartilages, but Nesbitt, Hunter and Bichat agree in opposing this opinion, and the author has in his museum a preparation distinctly shewing a portion of this membrane with its vessels stretching over the articular cartilage of a fœtal calf. The serous tissue, whether it assume the form of splanchnic membrane, synovial capsule, or mucous bursa, is composed of condensed cellular tissue, as may be easily ascertained by maceration. Its bloodvessels in a healthy state circulate colourless fluid; its lymphatics are so numerous that Mascagni was induced to assert that it was exclusively made up of them; but, from its almost complete insensibility in a healthy state, the nerves which supply it must be few and small. It is highly elastic but not contractile, it secretes an albuminous fluid, which is called synovia when exhaled by the bursæ mucosæ and synovial capsules. So long as this fluid is in moderate quantity the object is obtained for which it was intended; but if, from excessive action on the part of the exhalants or of diminished action on that of the absorbents, it accumulates, the functions of the organs it surrounds are impeded, and disease is produced. It has been fashionable with some to ascribe dropsy to increased exhalation alone, and to regard as unchanged and unchangeable the absorbent function; but facts are hourly occurring to prove that the healthy balance, which should subsist between these two systems of vessels, may be disturbed by the derangement of either, and that we may have dropsy as surely and as severely from absorbent atony as exhalent activity.

*Mucous and Cutaneous Tissues.* Under the same system the author comprehends the mucous and cutaneous tissues, and although some of their physical characters may appear dissimilar, their composition and functions shew them to be the same. If the fœtus be examined while an early embryo,

"There is so little difference between the external and the internal integument, that they are distinguished from each other with difficulty. The perfect continuity of the skin at the external apertures of the body with the internal membranes, is another proof which supports the opinion of these structures being identical. At the lips, at the nostrils, at the eyelids, at the anus, at the meatus urinarius, at the labia pudendi, we see the skin gradually changed into the mucous membrane. It is true, that around some of these openings a slight line of demarcation exists, as at the tarsal edge of the eyelids and at the lips; and a similar appearance may be more evidently observed in some of the internal parts of the body, which are contiguous with each other but distinct in function; as for example, where the cuticular lining of the œsophagus joins with the villous tunic of the stomach, and, in a less degree, in the connexion between the inner membrane of the vagina and the vascular coat of the uterus. But these distinctions do not extend to the *structure* of the two divisions of the cutaneous system, and therefore it cannot, with any truth, be asserted, that one is not perfectly continuous with the other.

"The organization of the animals which are placed lowest in the scale, displays, in a striking manner, the resemblance of the external and internal coverings of the body; in these creatures there are none of those diversities of appearance that have given rise to so much uncertainty in the human subject; the skin and the mucous membrane are, in fact, so completely identical, that one may, with impunity, be substituted for the other; so that if the polypus be turned inside-out, the new internal surface acquires the power of digestion, and the new external surface that of protecting the body. The effects of certain diseases, as for example, the prolapsus uteri and the prolapsus ani, occasionally shew that the mucous coat of the internal organs may become so altered by exposure to the air, as to assume in time some of the characters of skin." 175.

The skin is a complex organ consisting of, at least, three distinct laminæ, each of which is peculiar both in texture and office. The epidermis or external layer, Blumenbach says, can be again divided into several laminæ; Bichat has endeavoured to prove that it is perforated with pores which run in an oblique direction, and Chaussier supposes that the delicate filaments, which we see attached to its under surface after its separation from the cutis, are its exhalent and absorbent vessels. But Dr. Gordon regards these filaments as threads of pulpy matter formed by decomposition after death between the cuticle and skin; Chevalier has not been able, with the help of even the finest glasses, to detect any openings like distinct pores; and the author maintains that in its natural condition not the slightest appearance of laminæ is discoverable. It is the most extensive membrane of the body; not only covering its external surface, but entering by the natural apertures into the interior, and covering every part except the teeth which may be exposed to the external atmosphere, which induced Haller to observe that the teeth and the cuticle were the only organs which were allowed to come in contact with the air. It is deeply marked with linear depressions and corresponding elevations, which have a faint resemblance to the rugæ of some of the mucous membranes into which it is continued. These wrinkles are in part natural, to enable it to admit of more extended movements; partly, they are occasioned by the action of the subjacent muscles, and their size and number so increase with years that they are looked upon as a tolerably fair indication of either the age, or vigor of the constitution. Some have supposed that the cuticle is an inorganic substance, thrown out by the surface of the true skin; but when it is reflected that its reproductive power is greater than that of any other solid, that it is subject to disease, and that in scarlatina and other cutaneous affections it dies and exfoliates, we must conclude with the author that, like every other tissue of the human body, it is duly organized.

"The functions of the epidermis are so intimately connected with those of the skin in general, that only an imperfect account can be given of them in this place. Its most important use is to defend the body against the injurious influence of chemical and mechanical agents. It prevents the evaporation of the animal fluids in a very remarkable manner, so that a portion of dead skin, covered by the cuticle, may be exposed to the air for several weeks before it is dried; on the contrary, if the epidermis be removed, the cutis vera being deprived of its moisture soon becomes dry, hard, and discoloured. It moderates the impressions which are made on the nervous papillæ of the true skin, and guarding these delicate structures from the irritating contact of foreign bodies, it preserves that exquisite sensibility which is necessary for the proper exercise of the sense of touch. The complicated processes of cutaneous secretion and absorption are effected through the appropriate structure of the epidermis; and, lastly, the hairs, the nails, and the sebaceous glands, are all kept by it in their proper situations and offices." 198.

Between the epidermis and cutis Cruikshank succeeded in separating four distinct membranes; Gaultier has found three, others only one, and Bichat, Chaussier, and Rudolphi deny the existence of any. The author believes that in the negro there is a distinct membrane between the cuticle and cutis, which is the seat of color; and, although in Europeans he has never been satisfied of the fact, he presumes upon its existence in consequence of the various shades of color which their skin presents. Corresponding to the five varieties of the human species there are five leading varieties in the color of their skins. In the Caucasian it is more or less white; in the Mongolian it is yellow; in the Ethiopian black; in the American copper-coloured; and in the Malayan it is tawney. In Albinos there is neither pigmentum nigrum in the eyes, nor colouring membrane beneath the cuticle. Their skins, eyes and hair are, therefore, white, and the organic differences on which these peculiarities depend, by being transferable from parent to child, become hereditary. It has been popularly believed that the rete mucosum can never be regenerated when once destroyed; but Gordon and Meckel maintain the contrary, and Mr. Grainger inclines to the same view. Although neither blood-vessels nor nerves have been detected in it, the same reasons, which favor the organization of the cuticle, may be employed to shew that it also is organized. The colouring matter it contains seems principally to consist of carbon, and may be partly destroyed by maceration in water, or in a solution of chlorine. A negro made his foot nearly white by immersing it for some time in water impregnated with chlorine gas.

"The rete mucosum is considered by Mr. Chevalier as a second or internal epidermis, which serves as a delicate intermedium between the insensible cuticle and the vascular and nervous substance of the true skin; thus preserving the sensibility of the latter, and securing the regular performance of its numerous functions. It is also connected with the regulation of the temperature of the body, for it is itself a bad conductor of heat, and being placed immediately under another bad conductor and over a quick one, it must materially contribute to the uniformity of temperature, so necessary to an animal who is destined to inhabit all climates. The dark colour of the network of Malpighi in the African, has been for a long time considered as a provision of nature for the defence of the skin against the powerful effects of a tropical sun. This opinion is rendered more probable by the experiments of Sir E. Home. This distinguished physiologist ascertained that by tightly binding a piece of *black* kerseymere around his arm, a temperature, which burned off the nap of the cloth, produced no painful effect on the skin, although it was applied for fifteen minutes; on the contrary, when a piece of *white* kerseymere was similarly placed on the arm, a less degree of heat caused a blister in fifteen minutes. Sir Everard also found, that a temperature which excited pain and irritation in his own skin, produced none in the skin of a Negro.* We learn, however, from the observations of travellers, that the colour of the skin is not constantly in relation to the climate; but on the contrary, that there are dark races of people who inhabit the coldest regions, and light nations who live in the warmest climates; it is also known, that tribes entirely differing from each other in colour, inhabit the same latitude, and even the same islands.† Mr. C. Bell denies that the black skin preserves the Negro from the great heat of the African climate; he rests his opinion on the experiments of Priestley, which prove, that a dark body absorbs light and heat more rapidly than a white one, which repels them. But we should recol-

* "Phil. Trans. for 1821, p. 1, *et seq.*"

† " Dr. Prichard, Phy. Hist. of Mankind, vol. i. pp. 457 489.

lect that a high temperature irritates the skin, in consequence of acting on the surface of the cutis vera, and not on the rete mucosum, which is insensible. Now it is evident, that the latter, by absorbing the heat, prevents its action on the true skin; whilst in a white individual the heat passes through the semi-transparent epidermis and rete Malpighi, and thus acts immediately on the sensitive surface of the cutis. In support of Sir E. Home's theory, it may be mentioned, that Europeans who are exposed to the direct rays of the sun in hot countries, suffer severely from the irritation of the skin; at the same time that the dark inhabitants of these climates expose their naked bodies with perfect impunity."* 204.

The third and most essential lamina of the skin is the cutis vera, which, according to some is a muscular, according to others a fibro-cellular membrane. Its exterior surface, or *textus papillaris*, is covered with small elevations called papillæ, which are especially prominent upon the tongue. These papillæ are composed of nerves, blood-vessels, and lymphatics, are covered with rete Malpighianum and epidermis, to defend them from injury, and form the principal seat of the sense of touch. The interior surface is marked by depression or areolæ, which are perforated by many apertures, to transmit the hairs, nerves, and vessels of the skin. The reproductive power of the dermis is weaker than that of the epidermis, or rete mucosum; and when it is reproduced its supply of nerves and blood-vessels is diminished, its sensibility is decreased, and it never again discharges with the same effect the functions of the original skin.

The importance of the cutaneous tissue is, we believe, imperfectly understood; or, if understood, insufficiently attended to. After admitting it to be the special organ of sensation, it is looked upon by many as little more than a convenient envelope for defending more sensible organs from the stimulus of the air and other sources of excitement. But these uses, although the most obvious, are neither the only ones, nor the most important. The skin is both a respiratory and digestive organ. It inspires and expires as do the lungs, and precisely the same gases. The experiments of Cruikshank shew that carbonic acid is exhaled; and those of Abernethy that oxygen, if not azote, is inhaled. It has been long believed, though occasionally disputed, that it absorbs aqueous fluids; and there can be no doubt but that it secretes them. By placing frogs, toads and lizards in water it was ascertained by Edwards that their weight soon increased, and we have been ourselves the witness of experiments, which proved that a few minutes immersion either in cold or tepid water is followed by the same result in man. The importance of these facts is not yet sufficiently appreciated, partly because they are not universally admitted, and partly because when admitted they are not always seen in that practical aspect which to a careful observer they present. If the lungs, stomach and skin be organs of the same system, conducting functions of the same character, sympathizing with each other's state, bound up in one common interest, is it not evident that diseases affecting any one of them should be treated with a reference to the system of which it forms a part, and that the health and vigor of the whole can be secured only by attending to their individual welfare?

---

* "The above difference ought partly to be attributed to the much greater activity with which all the cutaneous functions are performed in the Negro."

The ultimate design of this community of office is obvious, but its immediate cause is obscure. Perhaps, in part it may be explained by the similarity of the tissues. The bronchial membrane of the lungs, the lining membrane of the stomach and intestines, and the external skin are essentially alike. They are all composed of cellular substance, they are all secreting and absorbing organs, they are all not only continuous, but convertible textures. The mucous system was arranged by Bichat into the gastro-pulmonary and genito-urinary divisions; but the ground-work of this division had been long before furnished by Bonn, who first demonstrated the continuity of the mucous membranes. The gastro-pulmonary division enters the body by the mouth and nostrils; the genito-urinary by the vulva in women and urethra in men. The nostrils and their appendages, the eustachian tube, cavity of the tympanum, and mastoid cells, the larynx, trachea and bronchi, pharynx, œsophagus, stomach and intestines are lined by the former; the urethra, vagina, uterus and its appendages, bladder, ureters and kidneys by the latter. When this lining membrane begins it is continuous with the external skin, when it ends it again becomes external integument; during part of its course through the interior of the body it is covered with a continuation of the external cuticle, and like the skin it varies in thickness according to the part it supplies, being finer in the sinuses of the head than in the bladder or intestines. In general the impressions of foreign bodies upon the mucous membrane are unperceived by the mind, but its natural sensibility is often augmented by disease, and where acute feeling is required, as in the lining membrane of the nose, it can exhibit great sensitiveness.

"In tracing the changes that occur in the vascular system, there are four principal points which it is desirable to determine: 1. The order according to which the different parts of this system first appear. 2. The arrangement which these parts observe at their primitive origin. 3. The relation which exists at different periods between the organs of the greater and lesser circulation. 4. The proportion between the number and capacity of the different orders of vessels in the several epochs of life.

"1. In attempting to ascertain what part of the vascular system is first developed, we are obliged to judge rather from the phenomena that occur in the process of incubation, than from any positive observations that have been made in man, or in the mammiferous animals. It may be stated, with tolerable certainty, that the veins of the *vesicula umbilicalis*, which in the human ovum corresponds with the membrane of the yolk, or *vitellary sack* of the incubated egg,* are the first which become apparent. The exact nature of the connexion that exists in the beginning, between the umbilical vesicle and the embryo, is rather obscure, but it is supposed that the cavity of the former communicates with the intestinal canal of the latter. It is evident that up to the second month the vesicle is connected by an artery and a vein, with the vessels of the mesentery; these communicating vessels are therefore called *vasa omphalo-mesenterica*.†

* "The analogy of the vesicula umbilicalis with the tunica erythroides, which is seen in the ova of some mammalia, was originally observed by Blumenbach. This umbilical vesicle has been frequently, but incorrectly compared to the allantoid membrane of quadrupeds."

† "A conjecture has been offered by Meckel which, in part, is opposed to the above description. He thinks it is probable, from the examination of the

"2. The vessels of the incubated egg first appear under the form of minute vesicles, or rather of rounded spaces which are formed in the vitellary membrane, and which are filled with a viscid fluid. These spaces are at first isolated and distinct from each other; but gradually increasing in number, they constitute a very ramified vascular network, which soon contains real blood. The minute vessels of this plexus unite, and at length form the trunk of the omphalo-mesenteric vein. These vessels, in their origin, do not possess distinct parietes; they are, in fact, merely canals hollowed in the substance of the yolk-bag; but insensibly this membrane becomes thickened around the canals, and thus forms the rudiments of their walls, which are progressively, but slowly, perfected.

"3. The omphalo-mesenteric vein being thus formed, passes from below upwards, and terminates in a vein which, ascending perpendicularly, is dilated at the point where the heart is ultimately formed. The aorta arises from this dilatation, and, by its branches, distributes blood to the different organs; the corresponding veins, and also the omphalo-mesenteric artery, appear nearly at the same time. It is most probable, that the umbilical vessels are developed in the same order as the omphalo-mesenteric, which they seem to supplant; that is to say, the vein first and the arteries afterwards." 262.

*Vascular Tissue.* The omphalo-mesenteric vein communicates with the vena portæ, which is at this period of growth the main trunk of the venous system. At its upper extremity an enlargement is observed corresponding with the situation of the heart; the left ventricle is first formed, then the aorta, and afterwards the auricle. The left ventricle and auricle are at first separate, but subsequently approximate into two contiguous cavities; the auricle divides into two apartments, communicating with each other by the foramen ovale; the right ventricle, which at first had the form of a small tubercle, is prolonged to the apex of the heart, and for a time communicates with the left, and last of all the pulmonary artery appears as a distinct vessel.

The muscularity of arteries has been and still is a warmly disputed point, and, although many experiments have been made and many arguments advanced to maintain the affirmative of the question, it is certain that many physiologists of the present day are yet dissatisfied. Haller and Bichat assert that arteries exhibit no manifestation of contractility when irritated; that their fibres move not when dissected layer by layer, that their nerves may be galvanized without effect, and that opium is inert when administered to them. All these arguments have been repeated by Craigie in defence of the same sentiment, and Nystem, in his "new galvanic experiments," could discover no symptoms of contraction in the arteries of a living dog, as in the human aorta. On the contrary Hunter, Thompson, Hastings, Phillips and Verschuir maintain that arteries, especially those of a small bore, may be made to contract when touched with acids and ammonia, when excited by galvanism, when irritated by the scalpel, or when exposed to the air. Bikker and Van-den-Bas by means of electricity, and Vassalli-Eandi Ginlio and Rossi, with the galvanic pile, arrived at the same conclusion. The author advances no new facts to *lay* this agitated question, or reconcile these conflicting evidences; but he strengthens the number of those who advocate the muscularity of arteries, by adding his voice to theirs in favor of their contractile power.

---

vascular system in acephalous monsters, and from the mode in which this system is formed in the animal kingdom, that the aorta is developed at the same time with the veins, or even before them."

It has been very generally believed that arteries are insensible, because when plied with irritating applications no pain was perceptible; but Mr. Grainger very properly observes—

"That several structures, which excite no sensation when touched, are yet exquisitely susceptible to the impression of their proper stimulants; thus the retina, which may be pricked with the cataract needle without the consciousness of the individual, is capable of being vividly impressed by the rays of light.* I conclude then that the arteries, and particularly their internal coat, are endowed with a peculiar sensibility, which is fitted to receive the impression of the blood, and that this fluid acts as the appropriate stimulus to the contractility of the fibrous tunic. The importance of this property in a pathological point of view, is illustrated by the interesting experiments that have been lately performed by Mr. Morgan and Dr. Addison, concerning the influence of poisons on the animal economy. These gentlemen appear to have determined the long disputed question, as to the *modus operandi* of these deleterious agents; by ascertaining that the noxious effects of poisonous substances, introduced into the current of the circulation, *do not result from their direct application to the brain itself, but from the impression made upon the sensible structure of the blood-vessels, acting upon the brain, through the medium of the nervous system.*"† 230.

*Osseous System.* In the early embryo the bones consist of a viscid fluid, which is supposed by some to be mucilaginous, by others gelatinous. The consistence of this fluid gradually augments; after some time it assumes the character of cartilage, and ultimately of bone. According to Beclard ossification commences about a month after conception, while Meckel extends the period to eight weeks. The long bones appear before the flat bones, and the flat bones before the short ones. The ossific matter may be deposited in three ways;—in a soft and gelatinous state, in the substance of cartilage, or between membranes. The pre-existence of cartilage is not necessary for the deposition of osseous matter, as was once supposed, for in the diaphyses of the long bones and in the centre of the broad ones horney particles are deposited ere cartilage has been formed. Du Hamel seeing that bones and trees had some resemblance to each other in general form, conceived that they might grow in a similar way. He fancied that periosteum was to the former what bark was to the latter, and that in process of time the one was converted into bone as the other was into wood. This doctrine was controverted by Haller, who, running into an opposite extreme, maintained that the periosteum had no concern in the formation of bone; that it was fabricated by vessels internal to its surface. Both of these views seem to be too confined, for both the periosteum and medullary membrane are concerned in conducting the ossific process. The experiments of Troja, Breschet and Villermé are quite decisive that in many instances the inner layers of the periosteum are ossified; and Cruveilhier has observed that

* "It is surprising how such an acute and admirable physiologist as Magendie, should have fallen into the error I have noticed above; such, however, is the fact, and in consequence of it, he is led into a contradiction concerning the retina: for, after stating that the central part of that membrane is more sensible than the rest of its extent, he goes on to assert that the sensibility of the retina may fairly be called in question. See Compend. of Phy. p. 47.

† "See Essay on the Operat. of Poisonous Agents upon the Living Body, p. 60."

when the medullary membrane has been wholly destroyed ossification proceeds at the surface of the bone, not only at the expense of the periosteum, but of the surrounding muscles. The same is shewn by the phenomena which occur during the re-union of a fracture. Blood is poured out by the lacerated vessels of the periosteum and neighbouring structure, and coagulates upon the broken ends of the bone. After a few days the red particles of the coagulum are absorbed, the periosteum swells and assumes by degrees the form of cartilage, osseous matter is now laid in this cartilaginous periosteum, as well as in the pale coagulum, and when the fractured parts are completely united the absorbents of the periosteum begin to act, the bony particles, which had been poured into it, are removed, and it ultimately returns to its ordinary texture.

*Muscular Tissue.* According to Prochaska muscular fibre, in whatever part of the body it be situated, or whatever length it may assume, does not exceed the $\frac{1}{40,000}$ part of an inch in diameter; it appears to be more or less flattened, or angular; to be solid and diaphanous. Sir A. Carlisle conceives it to be a solid cylinder, enveloped in reticulated membrane, and containing a pulpy substance irregularly granulated. M. Bauer and Sir E. Home find it to be made up of globules of the same size with those of the blood when deprived of their colouring matter, or $\frac{1}{2000}$ part of an inch in diameter. Dumas, Edwards and Dutrochet agree with Bauer and Home in asserting its globular structure, and the view is now very generally admitted. But Dr. Hodgkin, Messrs. Lister and Cooper and the author cannot acquiesce with any of these doctrines. With a microscope, having a magnifying power of 300, Messrs. Cooper and Grainger uniformly found "that an immense number of minute transverse lines could be perceived crossing the fibres, which were in some parts divided from each other by longitudinal lines apparently marking the lateral boundaries of the fibres; in most places, however, the small transverse lines were not separated from each other by any distinct division. Several large fibres were also seen crossing the lines in different directions, which appeared to consist of cellular tissue." 424.

The actions of muscular fibre are voluntary, involuntary and mixed; but no muscle can contract unless excited by some mental, mechanical, or chemical stimulus. This contractile power continues until death,—according to Beclard from 1 to 24 hours after it, and ceases in different muscles at different periods. Nystem informs us that it is first extinguished in the left ventricle, next in the intestines and stomach, then in the urinary bladder, afterwards in the right ventricle, next in the œsophagus, then in the iris, afterwards in the voluntary muscles, and lastly in the right auricle. The involuntary muscles are distinguished from those under the guidance of the will by consisting of fibres, which are not parallel with each other, but which interlace and inosculate, and which except the carneæ columnæ of the heart, do not possess any tendons. They are not remarkable for sensibility, since the heart has been touched while in full action almost without the consciousness of the individual. They are certainly not absolutely dependent upon the brain and nerves, since children have been born alive without either; yet from the influence which mental emotion and nervous irritation have upon their action, there can be doubt but that, connected with the nervous system as they are, the involuntary muscles must be considerably under their control.

*Nervous Tissue.* According to M. Serres the ganglia of the great sympathetic are formed before the spinal cord, the nerves of the trunk and pelvis grow independent of the brain, the spinal cord appears next, finally the brain, and the medullary portion is formed before that which is cineritious. This last point is much disputed. Gall maintains that the gray matter is formed first; Tiedemann asserts the contrary, and others believe that these two substances are at first intermixed in the form of a reddish white matter, and that they gradually separate as organization is matured. About the fifth week after conception a membranous tube is observed in the spine, having at its upper end a rounded pouch or vesicle, in which may be perceived, with the aid of a powerful lens, the rudiments of the two lateral columns of the spinal cord, and of certain parts of the brain. At the fifth month the anterior and posterior roots of the spinal nerves are seen, the cerebellum is beginning to separate into a central part and lateral lobes; at the seventh month these lateral lobes are more fully developed, and convolutions appear upon the cerebral hemisphere; and at the eighth month the various parts of the encephalon and spinal cord have received their perfect form, and sustain no subsequent alteration in any respect but size.

If the results of Della Torre can be depended on the nervous tissue consists of transparent globules, floating in a transparent but viscid fluid; which are smallest in the spinal cord, larger in the cerebellum, and largest in the brain. Prochaska denies that there is this difference in the size of these globules, or that they float in a fluid medium. He admits, however, that they do vary in magnitude, and that they are connected by a transparent cellular web. From a series of experiments performed by the two Wengels, they infer that the brain in mammiferous animals, birds, and fishes, consists of the same roundish bodies of which muscle, liver, spleen and kidney are composed, and that these bodies derive their shape from the cells of the cellular tissue. According to Sir E. Home and M. Bauer nervous matter is composed of globules, which present a fibrous appearance by being connected into lines by an intervening elastic jelly. The diameter of these globules varies from $\frac{1}{2400}$ to $\frac{1}{4000}$ part of an inch, the average size being $\frac{1}{3200}$. In the gray matter they are from $\frac{1}{3200}$ to $\frac{1}{4000}$ part of an inch in diameter, the smaller globules predominating; while in the white matter the large globules prevail. In the mesolobe the small globules were in great number, in the brain and peduncles of the cerebellum the quantity of intervening jelly prevailed over the globules, and when the nervous matter was immersed in alcohol all globular structure disappeared and the elastic jelly coagulated. The experiments of Edwards have led him to the same conclusions, except that he considers these globules of the same size whether they be in the brain or nerves of all the four classes of vertebrated animals; while Dr. Hodgkin and Mr. Lister have unsuccessfully looked for globular structure in either the nerves or brain.

"The source of the mysterious power, which operates through the medium of the nervous system, is entirely unknown; we are even ignorant of the manner in which this power acts on the material organs, the brain and the nerves, that are essential to the manifestation of its phenomena. The deficiency in our information has not resulted from any want of inquiry, for this question has at all times commanded an intense interest amongst philosophers. I shall only notice in this place the most modern, and at the same time the most plausible of the numerous theories which have been invented to remove the difficulty.

"It has been supposed by many eminent physiologists, amongst whom it will suffice to mention Cuvier, Abernethy, and W. Philip, that the power by which the nerves transmit impressions to and from the brain, is analogous to, or even identical with electricity. This hypothesis is strongly supported by the fact, that when a nerve is perfectly divided, its action may be imitated by galvanic electricity; thus, after the section of the par vagum, the secreting power of the lungs and stomach can be supported by galvanism. The evolution of heat from the blood, can also be accomplished by the same power; and it is well known that muscular contraction is susceptible of being excited by the agency of galvanism. It is likewise worthy of remark, that when the par vagum is simply divided, the nervous power is still transmitted to the stomach; and even when the two ends are separated to the extent of a quarter of an inch, a part of the nervous power is conveyed from the upper to the lower portion of the nerve. These last mentioned phenomena are very similar to those produced by electricity; and tend, in a very forcible manner, to shew the identity of the nervous and galvanic powers. But in establishing a doctrine of such importance, further evidence is required, and therefore it will be prudent, in the present state of the question, to defer forming any definite conclusion." 497.

The limits to which this review has been insensibly extended obliges us to bring it to a close; and the full analysis which has been given of Mr. Grainger's performance supersedes the necessity of adding any thing beyond a few general observations to what has been already written. In a work which is chiefly elementary much originality was not, perhaps, to be expected; and few subjects could occur either for controversy or criticism. Desirous of filling his pages with important matter, points of mere fancy have been generally avoided by the author; yet, in travelling a region which has been so imperfectly surveyed, it was impossible always to walk along a path of known and acknowledged safety. Unfrequented bye-ways must occasionally be trodden on; unascertained districts must sometimes be passed over. In such cases nothing safer can be done than to avail ourselves of whatever aid preceding travellers have left behind them; and, although the merit of discriminating faithful description from fanciful delineation be not equal to originality, it is often more than difficult to discover the best guides where the pretensions of the candidates are equalled only by their number. In general anatomy much is yet to be done; many points are utterly unknown, and very many are obscurely conceived. Mr. Grainger has in a plain and intelligible style presented his reader with a majority of the leading points which could be prudently relied upon in this extensive subject, where difficulties occurred they are faithfully pointed out, where doubts might be raised they are frequently anticipated, and when his own experience can shed any light upon the subject he is engaged in it is brought forwards willingly, but with an obvious consciousness that in questions surrounded with much difficulty to decide positively were more like presumption than philosophy. In this country such an elementary manual was much required, for hitherto our students have generally been driven to the alternative of remaining comparatively ignorant of a most interesting department of their professional literature, or of having to search the systems of France and Germany for instruction which they ought to have found at home.

